# Acute and Delayed Doxorubicin-Induced Myocardiotoxicity Associated with Elevation of Cardiac Biomarkers, Depletion of Cellular Antioxidant Enzymes, and Several Histopathological and Ultrastructural Changes

**DOI:** 10.3390/life11090880

**Published:** 2021-08-27

**Authors:** Alaa Abdelatty, Mohamed S. Ahmed, Mona A. Abdel-Kareem, Mohamed Dmerdash, Rehab Mady, Ahmed S. Saad, Ashraf Albrakati, Ehab Kotb Elmahallawy, Ahmed Elsawak, Walied Abdo

**Affiliations:** 1 Department of Pathology, Faculty of Veterinary Medicine, Kafrelsheikh University, Kafr Elsheikh 33516, Egypt; alaa_abdelatty@vet.kfs.edu.eg (A.A.); mohamed.abdelrahman1@vet.kfs.edu.eg (M.S.A.); ahmed.elsawak@vet.kfs.edu.eg (A.E.); waliedsobhy@yahoo.com (W.A.); 2 Anatomy and Embryology Department, Faculty of Medicine, Kafrelsheikh University, Kafr Elsheikh 33516, Egypt; Mona_abdelkarim2014@med.kfs.edu.eg; 3 Anatomy Department, Faculty of Medicine Al-azhar University, Cairo 11884, Egypt; drdmerdash@gmail.com; 4 Department of Pharmacology, Faculty of Veterinary Medicine, Damanhour University, Damanhour 22511, Egypt; Rehabmady1976@gmail.com; 5 Department of Pharmacology and Toxicology, Faculty of Pharmacy, Port Said University, Port Said 42526, Egypt; mosa1200@yahoo.com; 6 Department of Human Anatomy, College of Medicine, Taif University, P.O. Box 11099, Taif 21944, Saudi Arabia; a.albrakati@tu.edu.sa; 7 Department of Zoonotic Diseases, Faculty of Veterinary Medicine, Sohag University, Sohag 82524, Egypt

**Keywords:** acute, delayed, myocardiopathy, doxorubicin, antioxidant enzymes

## Abstract

Doxorubicin (DOX; Adricin) is an anthracycline antibiotic, which is an efficient anticancer chemotherapeutic agent that targets many types of adult and pediatric tumors, such as breast cancer, leukemia, and lymphomas. However, use of DOX is limited due to its cardiotoxic effects. This study sequentially investigated the mechanistic pathways of the cardiotoxic process of DOX in rats at different post-treatment periods using cumulative dose, which is used in therapeutic regimes. In this regard, 56 male albino rats were used for the experiment. The experimental animals were divided into seven groups (*n* = 8/group) based on dose and sacrifice schedule as follows: G1 (2 mg/kg body weight [BW] and sacrificed at day 4), G2 (4 mg/kg BW and sacrificed at day 8), G3 (6 mg/kg BW and sacrificed at day 15), G4 (8 mg/kg BW and sacrificed at day 30), G5 (10 mg/kg BW and sacrificed at day 60), G6 (10 mg/kg BW and sacrificed at day 90), and G7 (10 mg/kg BW and sacrificed at day 120). As expected, G1, G2, and G3-treated groups revealed features of acute toxic myocarditis associated with degenerative and necrotic changes in myocytes, mitochondrial damage, elevation of cardiac biomarkers, and depletion of cellular antioxidant enzymes. However, these changes increased in severity with subsequent treatment with the same dose until reaching a cumulative dose of 10 mg/kg BW for 30 d. Furthermore, after a cumulative dose of 10 mg/kg BW with a withdrawal period of 2–3 months, various predominant changes in chronicity were reported, such as disorganization and atrophy of myocytes, condensation and atrophy of mitochondria, degranulation of mast cells, and fibrosis with occasional focal necrosis, indicating incomplete elimination of DOX and/or its metabolites. Altogether, these data provide interesting observations associated with the cardiotoxic process of DOX in rats that would help understand the accompanying changes underlying the major toxic effects of the drug. Future research is suggested to explore more about the dose-dependent mechanisms of such induced toxicity of DOX that would help determine the proper doses and understand the resulting cardiomyopathy.

## 1. Introduction

Doxorubicin (Adricin) is an anthracycline antibiotic isolated from *Streptomyces peucetius var. caesius* and is an efficient anticancer chemotherapeutic agent that targets many types of adult and pediatric tumors, such as breast cancer, leukemia, and lymphomas [1,2]. The mechanism underlying the anticancer effect of doxorubicin (DOX) has been proposed to involve inhibition of DNA and RNA synthesis; thus, preventing the replication of rapidly proliferating cancer cells [3,4,5,6]. However, use of DOX in treating different tumor types is limited due to its numerous acute or chronic side effects [7,8], mainly cardiotoxicity, which may lead to fatal congestive heart failure and cardiomyopathy, even after a long period from treatment cessation, among other side effects [6,9]. Reviewing the available literature, the evidence of myocardiopathy of DOX was elucidated through an elevation of serum cardiac enzymes as AST, LDH and CK–MB enzymes. Among these biomarkers, cardiac troponins have been considered the most sensitive one for myocardial injury [10]. Clearly, these cardiac biomarkers could be used as non-invasive indicators of the oxidative stress accompanying the myocardial injury since they are extremely elevated with severe injury including ischemia and infarction [11]. 

The mechanism of cardiotoxicity resulting from DOX has been documented in various previous studies [6,12,13]. Several mechanisms have been proposed, such as generating reactive free radicals, leading to increased lipid peroxidation, and oxidative damage of cardiac muscle [14,15]. Furthermore, the activation of apoptotic signaling with subsequent cardiomyocyte apoptosis has been suggested [15,16,17,18,19]. Additionally, the morphological features of DOX-induced cardiomyopathy have been described, mainly in rats [20,21,22,23]. Some authors have reported acute changes using high doses or described changes shortly after the last treatment [18,24,25,26], while others described delayed chronic alterations [27,28]. However, limited information is available about the cardiotoxicity of DOX in rats at different post-treatment periods using cumulative doses in therapeutic regimes. Given the lack of information in this context, the present study sequentially investigated the cardiotoxic process of DOX in rats at different post-treatment periods using cumulative dose, as used in humans. We also correlated morphological alterations in the myocardium and activity of serum CK–MB to better understand the possible mechanistic pathways involved in the cardiotoxicity of DOX.

## 2. Materials and Methods

### 2.1. Materials

#### 2.1.1. Ethical Considerations

The study protocol was carefully reviewed and approved by the local guidance of Research, Publication, and Ethics of the Faculty of Veterinary Medicine, Kafrelsheikh University, Egypt, which complies with all relevant Egyptian legislation. The ethical approval number is KFS-2018/4.

#### 2.1.2. Animals

Fifty-six male albino rats (weighing approximately 120–150 g) were used in our experiment. Rats were housed in metal cages and acclimatized to laboratory conditions for two weeks. The animals were provided with a commercial balanced diet and tap water *ad libitum* throughout the experiment.

#### 2.1.3. Chemicals

Adricin (Doxorubicin HCl) 25 mL solution of 50 mg doxorubicin HCl was purchased from EIMC United Pharmaceuticals Company (Badr city, Cairo, Egypt). The drug was stored at 4 °C and protected from light. A new vial was used with each injection.

### 2.2. Methods and Experimental Design

#### 2.2.1. Experimental Design and Schedule of Sacrifice

**Figure 1:**
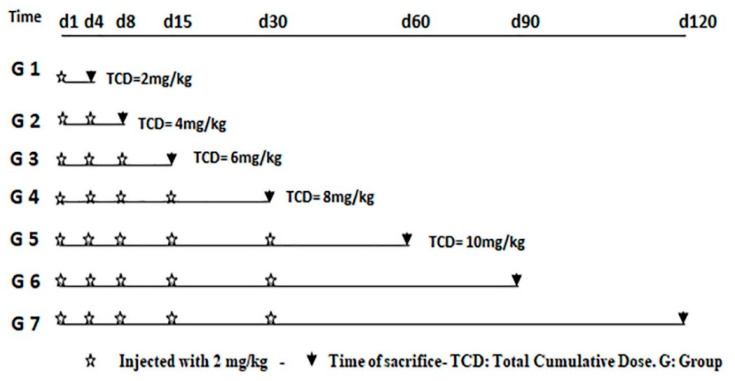
Experimental protocol used in this study.

The rats were divided into seven groups (G1–G7), each consisting of eight animals. Three animals were treated with intraperitoneal saline injections (control animals), and five animals were intraperitoneally injected with DOX (treated animals). Animals received repeated intraperitoneal injections of 2 mg/kg body weight DOX to reach a total cumulative dose of 10 mg/kg, as recommended in the 5th, 6th, and 7th groups as described in another study [29], for two reasons. The first was that 10 mg/kg DOX induced a similar degree of cardiac dysfunction as 15 mg/kg but induced significantly less diarrhea and ascites, and enhanced survival rates. The second reason was that 10 mg/kg is equivalent to the cumulative toxic dose in humans, 550 mg/m^2^. Additionally, the intraperitoneal route of administration of the drug is considered to be the best injection route to avoid severe perivasculitis and necrosis resulting from intravenous injection in the tail vein [30]. Animals were euthanized under ether anesthesia by decapitation. The schedule of injections and sacrifices is summarized in Figure 1.

#### 2.2.2. Blood/Serum Collection and Analysis

Orbital blood samples were collected using microcapillary tubes under light ether anesthesia just before sacrifice. Approximately 3 mL blood was collected from each animal. Serum was separated from clotted blood by centrifugation at 3000 rpm for 15 min and stored at −20 °C until use. 

#### 2.2.3. Serum Biochemical and Antioxidant Analysis

The serum levels of the cardiac biomarkers, including Aspartate Aminotransferase (AST) and CK, were quantified according to the available standard diagnostic kits (Stanbio Laboratory, Boerne, TX, USA). Additionally, the serum cardiac troponin T (cTnT) concentration was assessed using an ELISA kit (Roche Diagnostics, Mannheim, Germany). Finally, the cardiac tissue homogenates were used to estimate the following markers: catalase (CAT, Biodiagnostic, #CA 2517, Dokki, Giza, Egypt), superoxide dismutase (SOD, Biodiagnostic, #SD 2521, Dokki, Giza, Egypt), and glutathione peroxidase (GPx, Biodiagnostic, Dokki, Giza, Egypt) using commercial test kits obtained from Biodiagnostic Company (Giza, Egypt).

#### 2.2.4. Histopathological and Ultrastructural Examination

##### Histopathological Examination

Heart tissue sections, including ventricular portions of each animal, were collected according to the planned schedule. First, the sections were directly fixed in 10% formalin solution. Next, the specimens were dehydrated in alcohol and cleared in xylene, and embedded in paraffin wax. Then, blocks were sectioned in 4 µm thickness and stained using hematoxylin and eosin (H&E).

##### Ultrastructural Examination

Heart specimens from the left ventricle from different treated groups were fixed in 3% glutaraldehyde in 0.1 M sodium cacodylate buffer (pH 7.4) at room temperature for 24 h, then washed overnight in several changes of 0.1 M sodium phosphate buffer (pH 7.4) at 4 °C. Later, the samples were post-fixed in 2% osmium tetroxide in 0.1 M sodium phosphate buffer at room temperature. Specimens were dehydrated in cold ethanol and propylene oxide in ascending grades and then embedded in Spurr’s resin. Ultrathin sections (50 nm) were prepared and subsequently double-stained for 10 min with 3% aqueous uranyl acetate and 10 min in lead citrate. Samples were then observed under aJEM, 100CXII electron microscope, Assuit University.

#### 2.2.5. Statistical Analysis:

Statistical analysis was performed using GraphPad PRISM software v.5 (La Jolla, CA, USA). Data were analyzed and compared with One-way ANOVA and Tukey’s post hoc multiple range tests. Statistical significance was accepted at *p* < 0.05.

## 3. Results

### 3.1. Doxorubicin Induced Sgnificant Changes in Biochemical and Antioxidant Enzymes

In this study, DOX induced significant AST, CK–MB and cTnT serum activity changes in comparison with their corresponding groups-specific control. These enzymes were gradually elevated in rats after receiving DOX at doses of 2, 4, or 6 mg/kg body weight and reached the maximal level after exposure to 8 mg/kg body weight. The levels of cardiac biomarkers decreased in withdrawal groups. However, enzyme levels were still increasing in comparison with groups-specific control (Table 1). Comparing the cardiac enzymes profile of withdrawal groups with G5-treated group, only cTnT biomarker showed significant decrease in G6-treated group. On the other hand, the remaining examined biomarkers demonstrated non-significant decrease of serum AST and CK–MB levels. Furthermore, DOX-treated animals sacrificed at 4 months (G7-treated) showed significant decrease of biomarkers in comparison with G5-teated group (Table 1). Additionally, cardiac antioxidant enzymes were also markedly altered. A significant decrease in tissue CAT, SOD, and GPx was noticed with acute and delayed DOX therapy. The decrease in these enzymes was noticed dose-dependently until G5-treated showed a marked decrease in these biomarkers. An increase of CAT antioxidant level was noticed in of the early withdrawal group (G6-treated) in comparison with G5-treated, and a noticeable elevation of the antioxidant enzymes of late withdrawal group (G7-treated) was significant over that in G5-treated. However, these levels did not return to the normal limits of the control-specific group (Table 1).

### 3.2. Light Microscopic Examinations of Cardiac Myocytes

**Figure 2:**
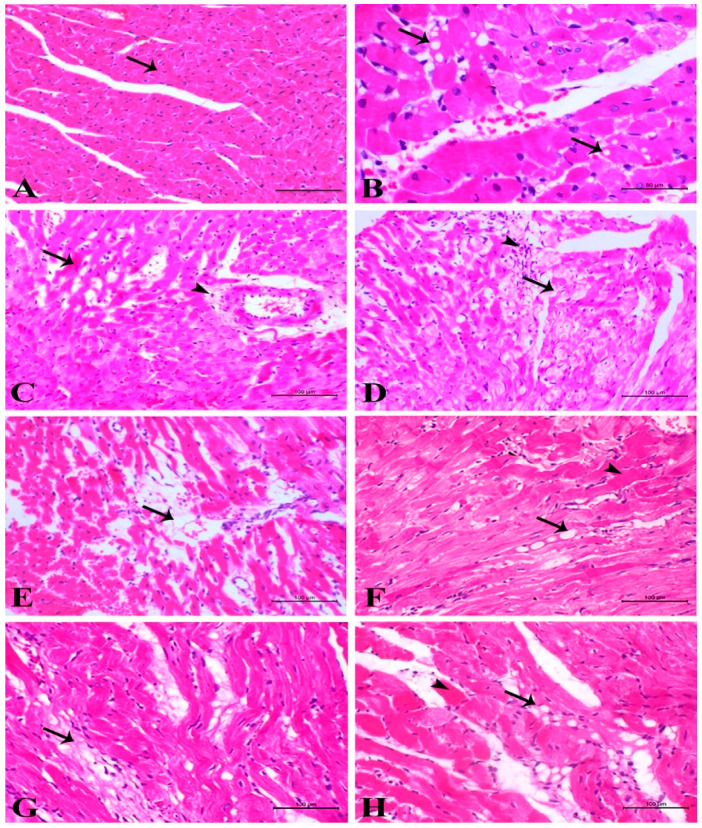
(**A**) Control heart showing normal myocyte (arrow). (**B**) G1-treated showing myolysis with loss of striation and sarcoplasmic vacuolation (arrows). (**C**) G2-treated showing sarcoplasmic eosinophilia with perivascular edema associated with vascular endothelial retraction. (**D**) G3-treated showing sarcoplasmolysis (arrow) and occasionally focal fibroplasia (arrowhead). (**E**) G4-treated showing marked complete focal myolysis indicated by acellular areas (arrow) with severe perivascular and interstitial edema. (**F**) G5-treated showing diffuse coagulative necrosis (arrowhead), sarcoplasmolysis, and both sarcoplasmic and interstitial vacuolation (arrow). (**G**) G6-treated showing multifocal areas of fibrosis with cardiomyocyte atrophy (arrow). (**H**) G7-treated showing necrosis of the muscle fiber (arrowhead) and disorganization and atrophy of myocardiocytes with interstitial fibrosis (arrow); (H&E, bar = 100 µm).

This step included examining the cardiac myocytes, blood vessels, and myocardial interstitium in the left ventricle, where the more advanced changes in cardiac myocytes were mainly observed in perivascular and subendocardial areas. Thus, the examination of control animals in all groups revealed normal features of the myocardium (Figure 2A). However, examination of the heart section of G1-treated revealed eosinophilic sarcoplasm of cardiac myocytes accompanied by loss of striation mostly because of damage to contraction banding. At some sites, sarcoplasmic vacuolation was observed, indicating the dilatation of sarcotubules and terminal cisternae by occasional loss of myofibrils and the beginning of myolysis (Figure 2B). Areas of obvious coagulative necrosis of cardiac myocytes with active myophagia and slight to moderate mononuclear cell infiltration, giving features of focal acute toxic myocarditis, were also found. With previously described parenchymal changes, vascular changes were frequently observed, particularly dilatation of blood capillaries. This dilatation was advanced at some sites, causing a disturbance of the myocardial architecture and produced a syncytial arrangement of myocardiocytes. In addition, most blood vessels revealed retracted and swollen endothelial cells associated with perivascular edema (Figure 2C). The changes after the second treatment did not differ in principle from the observed changes after the first dose. Sarcoplasmic eosinophilia with loss of striation, coagulative necrosis of cardiac myocytes, and vascular endothelial changes with perivascular edema were present, while the vacuolation of sarcoplasm was not always evident. The most striking change was perivascular and subendocardial coagulative necrosis of cardiac myocytes. After the third treatment, the parenchymal and vascular changes were not greatly different from those observed after the first or second treatment but with certain sarcoplasmic vacuolations. However, perivascular and subendocardial coagulative necrosis of cardiac myocytes was frequently observed. In perivascular areas, necrotic changes were accompanied by inflammatory cell reaction, presenting features of toxic myocarditis with marked perivascular edema (Figure 2D). Furthermore, a focal and diffuse interstitial cell reaction could be identified in this group.

The changes in myocardiocytes were more diffuse in G4-treated. Sarcoplasmic eosinophilia, loss of striation, less cytoplasmic vacuolation, and coagulative necrosis were observed along with sarcoplasmolysis, evidence of cardiac myocyte atrophy, and occasional beginning of focal fibroplasia. Additionally, perivascular and interstitial edema was predominant. However, the big acellular areas can represent complete lysis and disappearance of some cardiac myocytes (Figure 2E). Regarding G5-treated, the damage to myocytes was diffuse, and either coagulative necrosis or sarcoplasmolysis with discrete vacuoles sometimes enclosed the nuclei. Complete myolysis was also evident. Additionally, interstitial and perivascular edema was obvious and accompanied by a certain degree of interstitial cell reaction and fibroplasia, while fibrosis was observed in perivascular areas (Figure 2F). However, the predominant picture in G6 showed disorganization of myocardial architecture, focal fibrosis, and atrophy of cardiac myocytes. A few areas revealed sarcoplasmolysis, coagulative necrosis with a typical picture of myophagia, and interstitial cell activation (Figure 2G). The lesions in G7-treated did not differ from those observed in G6-treated, and disorganization, fibrosis, and atrophy were the main observed changes. However, occasional foci with discrete myocytes with coagulative necrosis or sarcoplasmolysis were still present, while several vacuoles were observed in the interstitium, which is mostly fat (Figure 2H).

### 3.3. Electron Microscopic Examinations of Cardiac Myocytes and Their Mitochondria

**Figure 3:**
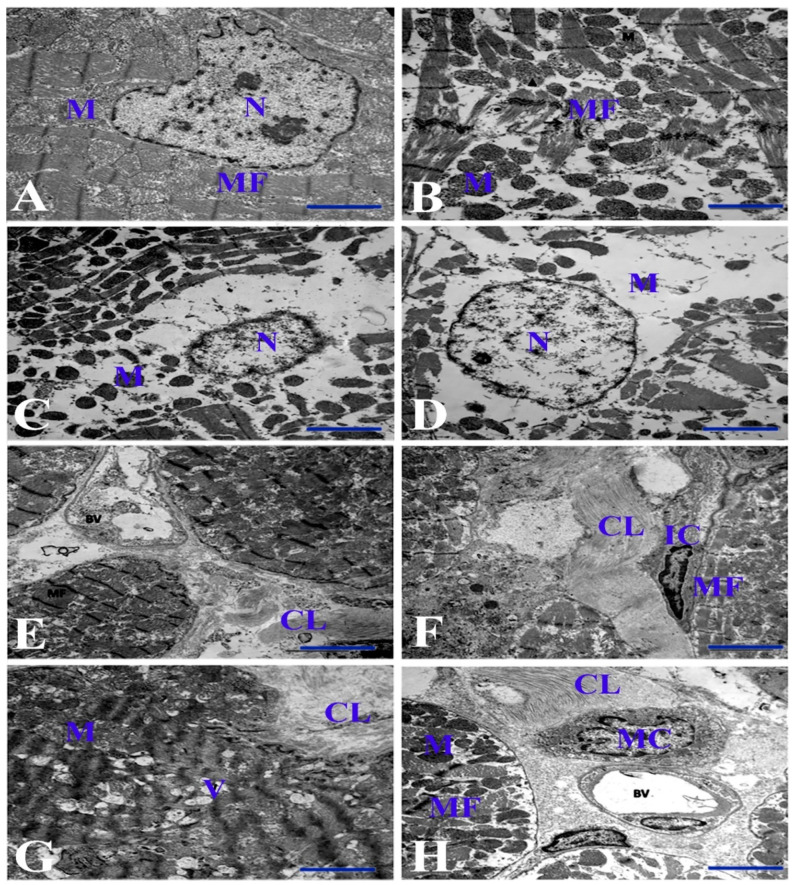
Electron micrograph of the cardiac muscle of different groups. (**A**) Control heart showing normal myocyte. (**B**) G1-treated showing myofibrillar disorganization and marked myofilament lysis. (**C**) G2-treated showing marked perinuclear myofilamental lysis. (**D**) G3-treated showing a severe degree of myolysis. (**E**) G4-treated showing disorganized myofibrils and bundles with marked collagen fiber proliferation separating two myocytes. (**F**) G5-treated showing bundles of collagen fiber interposed by interstitial cells. (**G**) G6-treated showing advanced interstitial fibrosis exemplified by large bundles of collagen fibers and perivascular edema with distortion of some myofibrils, occasional sarcoplasmic vacuolation, and severe mitochondrial damage. (**H**) G7-treated showing perivascular edema and fibrosis associated with the presence of degranulated mast cells. Bar = 2 µm. N = nucleus. MF = myofibrils. M = mitochondria. CL = collagen fibers. IC = interstitial cell. V = vacuole. BV = blood vessel. MC = mast cell.

Electron microscopy of control animals in all groups revealed normal ultrastructure of the myocardium (Figure 3A). However, myocytes in G1-treated showed varying degrees of degenerative changes ranging from myofibril disorganization to myofilament lysis and loss, leaving rarefaction inside myofibrils. A big gap was also found in the interfibrillar matrix filled with myofilament debris. The mitochondria showed slight degenerative changes with disorganization. The changes were dilatation of mitochondrial cristae and occasional damage to the inner membrane with matrix lysis (Figure 3B). The nuclei revealed chromatolysis, but with an intact nuclear membrane. The changes in G2-treated were similar to those in G1-treated, but more extensive. The myocytes revealed large areas of disorganized myofibrils and myofilamental lysis, and the sarcoplasm contained a great amount of myofilamental fragments, while the mitochondria revealed more disorganization and vacuolation (Figure 3C). The ultrastructural features in G3-treated were similar to those in G2-treated, but some mitochondria became condensed, and the nucleus was distorted (Figure 3D). In G4-treated, some collagen fibers separated two myocytes. Moreover, the myocytic features revealed disruption and lysis of myofilaments, while the mitochondria showed severe disorganization, destruction, vacuolation, and atrophy, particularly at the nucleus pole. The nucleus showed distortion with unusual contour appearance with disorganization of chromatin pattern and disrupted nuclear membrane (Figure 3E). Additionally, an area of early and complete lysis of myofibrils with sarcoplasmic vacuolation was observed in this group. G5-treated was characterized by the presence of many intracellular vacuoles of varying sizes. Probably these vacuoles are dilated sarcoplasmic reticula and can be partly related to the lysis of myofibrils (Figure 3F). Furthermore, a big area of fibrosis was represented by bundles of collagen fibers interposed by fibroblasts. The mitochondria at the nuclear pole showed severe destruction and became highly condensed, while the nuclei revealed a bizarre shape and showed disorganization and clumping of chromatin.

The ultramicroscopic features of G6-treated were close to those found in G5 with prominent advanced fibrosis. Some myofibrils revealed distortion due to myofilament lysis with occasional sarcoplasmic vacuolation. The mitochondria were severely damaged and decreased in number in areas of both nuclear poles. Additionally, atrophy and condensation were observed in interfibrillar spaces. Advanced interstitial fibrosis was exemplified by bundles of collagen fibers running between the myocytes, and perivascular edema was evident (Figure 3G), while the nucleus was bizarrely shaped with peripheral chromatolysis. In G7-treated, some fibrils showed a certain degree of degeneration, disorganization, and lysis or atrophy, while the mitochondria were more condensed and atrophied. The nucleus showed nearly normal features with chromatin clumping, thin regular condensed chromatin at the nuclear periphery, and normal contour and nuclear membrane. Perivascular edema and fibrosis, indicated by the presence of collagen fibers, were evident and associated with the presence of degranulated mast cells (Figure 3H).

## 4. Discussion

To the best of our knowledge, DOX causes dose-dependent selective cardiotoxicity [31,32,33]. Various mechanisms have been suggested for the resulting cardiotoxicity after exposure to DOX. Some previous studies have proposed a high cardiac susceptibility to a much lower concentration of enzymatic defenses by SOD1 and CAT against free radical attack and a sustained drug-related depression in cardiac glutathione peroxidase activity following exposure to DOX [34,35]. Furthermore, DOX decreases the protein levels and activity of SOD1 [36]. Given that mitochondria are abundant in cardiac tissue (approximately 35% of the cell volume), it has been suggested that the primary mechanism for cardiotoxicity is mitochondrial dysfunction, possibly via interference with calcium homeostasis [37,38,39]. The mitochondria-related mechanism relies upon ATP to sustain contractile function. Consequently, interfering with this function is likely to cause cardioselective toxicity [38]. This study provides interesting histopathological findings and features of toxic myocarditis following examination of DOX-treated rats. These features appeared to be acute after exposure to doses of 2, 4, or 6 mg/kg body weight, and there were no significant differences after exposure to these doses. The pathological features appeared to be chronic after exposure to doses of 8 or 10 mg/kg body weight. Examination of the heart ventricles of nearly all treated rats revealed perivascular and subendocardial coagulative necrosis and contraction band degeneration. More importantly, these myocytic changes were confirmed by electron microscopy, which revealed varying degrees of myofibrillar degenerative changes ranging from myofibrillar disorganization to myofilament lysis and loss. In addition, mitochondria revealed relatively slight degenerative changes, disorganization, and vacuolation in acute toxicity with smaller doses and after a short period following DOX exposure. These changes became more extensive in the chronic toxicity study, wherein mitochondria showed severe destruction, condensation, and atrophy. DOX also caused vascular changes, such as the dilatation of blood vessels and perivascular edema, which were frequently observed. Fibrosis was observed beginning from G5-treated where rats received the total cumulative dose and were sacrificed one month after the last dose.

As shown in this study, the myocardial degenerative changes were dose- and time-dependent. The lesions increased with increasing dose and time from the last DOX administration until G5-treated. Similar time dependency was reported after two and four weeks from the exposure to 15 mg/kg body weight [40]. The authors found more extensive degenerative changes after four weeks. Additionally, a dose-dependent effect of DOX on tissue culture was recorded, wherein larger doses induced more apoptosis and left less viable cells than smaller doses [24,41]. Sarcoplasmic eosinophilia, loss of striation, and obvious coagulative necrosis of myocytes were the most prominent changes observed by light microscopy in the acute toxicity group in this work, and these lesions were mostly perivascular and subendocardial. In contrast, rats exposed to 8 mg/kg body weight with withdrawal time two weeks (G4-treated) or 10 mg/kg body weight with withdrawal time four weeks (G5-treated) revealed diffuse changes accompanied by sarcoplasmolysis and cardiac myocyte atrophy. In rats exposed to 10 mg/kg body weight with withdrawal time of two or three months (G6-treated and G7-treated), only a few areas revealed sarcoplasmolysis and coagulative necrosis with disorganization of myocardial architecture. These findings agree with what has been described after five months from the last treatment of a total cumulative dose of 10 or 20 mg/kg body weight [30]. In the same study, the authors described focal myocyte degeneration in the form of densely eosinophilic cells lacking cross-striations [30]. The presence of such areas of necrosis after a long withdrawal time indicates persistent exposure to the toxin [42,43]. This might be attributed to the slow excretion rate and prolonged clearance of DOX and its metabolites from the body [44].

The presence of slight to moderate focal mononuclear cell infiltration in the first three groups indicated features of focal acute toxic myocarditis. The electron micrographs confirmed the previous changes, where the presence of large areas of myofibrillar disorganization, lysis, and loss with interfibrillar matrix filled with myofilament debris characterized the three groups with acute toxicity. The severity of the previous changes slightly decreased in the following groups, but they were present. Myofibrillar disorganization, lysis, and loss have been previously described by several authors [45]. In a previous study, the use of DOX at a dose of 3 mg/kg body weight weekly for four weeks and sacrifice of the rats five weeks after the last treatment caused randomly distributed damaged myocytes [45]. Induction of chronic toxicity in mice using a dose of 1 mg/kg body weight weekly for 12 weeks and sacrificing them one week after the last injection produced a marked disruption of the myofibrillar array, leading to focal loss of banding in myocytes [28]. However, rats exposed to 2 mg/kg DOX once a week for seven weeks and sacrificed one week after the last injection revealed distributed myofibrillar loss [46]. The difference in the degree of myofibrillar damage and its nature in this study and what was recorded by these authors may be related to differences in the cumulative dose and the sequential sacrifice in this study. Additionally, it was mentioned that the myofibrillar loss probably results from DOX interference with protein synthesis [47].

It should be emphasized that the damage of contracting bands, coagulative necrosis, and myofibrillar lysis was mostly related to active oxygen species, which were produced through the activation of endothelial nitric oxide synthesis or NADPH enzyme activation in DOX-cardiotoxicity [34,48,49,50]. It was also hypothesized that DOX induced cardiotoxicity and myocyte damage through lipid peroxidation due to an increase of both mitochondrial iron and cellular ROS levels [51,52,53,54]. Enzyme markers, AST, CK–MB, and cTnT, are the most widely used biomarkers that indicate suspected myocardial injury caused by DOX [34]. The magnitude of their activity in serum after myocardial injury reflects the extent of damage to the musculature. In this study, DOX caused significant changes in the serum activity of these biomarkers that were parallel to the degenerative changes observed in each group. It should be noted that these changes appeared to be related to both the total cumulative dose and withdrawal time of DOX. In this regard, cardiac enzyme serum activity gradually elevated after rats received DOX doses of 2, 4, or 6 mg/kg body weight and reached the maximal level after exposure to 8 mg/kg body weight. Then, enzyme activity gradually declined after exposure to 10 mg/kg body weight. This decline reached its nadir in animals with withdrawal time three months after the last dose. These results agree with a previous study of a total cumulative dose of 12 mg/kg body weight and withdrawal time of 6–9 weeks [55]. Interestingly, light microscopy revealed sarcoplasmic vacuoles of varying size in the first five groups. Similar vacuoles were recorded after using doses of 1 mg/kg body weight and 2 mg/kg body weight for ten injections to reach 10 and 20 mg/kg body weight as total cumulative doses, respectively [30]. Additionally, similar vacuoles appeared after exposing the rats to 3 mg/kg body weight for four weeks, followed by sacrifice five weeks after the last injection [45]. Thus, the absence of sarcoplasmic vacuolation in this study in the last two groups may be due to the long withdrawal period (two and three months). More importantly, in our work, these vacuoles were also viewed by electron microscopy till G6-treated and were similar to those recorded in previous investigations [27,30,45,46]. Some previous studies have proposed that these vacuoles were probably dilated sarcoplasmic reticula and terminal cisternae attributed to redistributed intracellular water and electrolytes [28,30,56]. Furthermore, our study reports a clear perinuclear space by electron microscopy, which might represent the lysis of perinuclear myofibrils and disarrangement of perinuclear mitochondria. This observation agrees with previous reports [57].

As depicted in our results by light microscopy, the total cumulative toxic dose (10 mg/kg body weight) induced fibrosis in the last three groups, which was more obvious in G6 and G7-treated. This induced fibrosis is represented by collagen bundles between the myocytes in electron micrographs. This latter finding was confirmed in the last three groups by staining heart sections from the chronic groups with trichrome stain showing interstitial and perivascular fibrosis, which was prominent. Additionally, features of atrophic myocytes and myofibrils were clearly observed. In a previous study, clear collagen fibers between myocytes were observed after exposure of rats to DOX at a dose of 3 mg/kg body weight daily for four days with sacrifice after 15 days from the beginning [56]. The authors in the same study mentioned that fibrosis might result from lipid peroxidation as DOX could stimulate collagen synthesis. Several experimental and clinical studies have already shown the relationship between fibrosis and lipid peroxidation [58,59,60,61]. It was reported that oxidative reactions directly stimulate procollagen type 1 gene expression, contributing to fibrosis development [58]. Similarly, fibrosis was described after treating rats with DOX at 1.5 mg/kg body weight weekly for eight weeks with sacrifice 6–9 weeks after the last injection [55]. Edema, either perivascular or in the interstitium, where it was a general feature, was accompanied by vascular changes in the form of dilated blood vessels and retracted endothelial cells after exposure to all doses. Similar lesions were described in previous studies [30,57,62,63]. Given the hypothesis that edema resulted from a disturbance in cardiac electrolytes, DOX was reported to increase sodium content and calcium in the cardiac ventricle during the induced cardiotoxicity in rabbits [64]. As shown in our results, the mitochondria revealed a dose-dependent effect where 2 mg/kg body weight caused slight degenerative changes. These changes were revealed in electron micrograph in dilatation of the mitochondrial cristae with damage to the inner membrane and lysis of their matrix. A previous study referred to the injury of mitochondria as the result of retention of the cationic adriamycin in the mitochondrial inner membrane forming an irreversible complex with cardiolipin, which is an inner mitochondrial protein [65]. Similar but more extensive changes with vacuolation were observed after exposure to a cumulative dose of 4 mg/kg body weight, while the larger cumulative doses of 6, 8, and 10 mg/kg body weight made the mitochondria more condensed with severe disorganization, destruction, and atrophy. Similar mitochondrial disorganization and lysis of their matrices have been reported [28]. Similar findings were also recorded 10 days after exposure to a single dose of 20 mg/kg body weight [66]. The effect of DOX on the respiration of submitochondrial preparation was studied, and it was proved that DOX produces reactive oxygen species in the presence of NADH [67]. A previous report mentioned that the mitochondrial structures were impaired due to resulting free radicals from the effects of doxorubicin that caused lipid peroxidation in mitochondrial membranes [56]. Additionally, it was concluded that adriamycin in the presence of iron strongly binds to submitochondrial particles, causing inhibition of their respiratory enzymes [68]. The increase in the volume of the mitochondria after exposure to 2 and 4 mg/kg body weight was probably a compensatory response of the cell to supply the energy needed for all reactions involving the cell’s metabolic activity [56]. The high density of mitochondria may be caused by increased matrix volume and a concomitant decrease in the intermembrane compartment [69].

Additionally, DOX produced mutation in mitochondrial DNA more than nuclear DNA in cardiac tissue [70]. However, this study proved that DOX causes nuclear damage even with a small dose: the most obvious nuclear changes were disorganization and clumping of chromatin, chromatolysis and unusual contour, and destruction of the nuclear membrane. A similarly disorganized nucleus with a fragmented nuclear membrane was previously observed [40], and these nuclear changes probably result from the binding of DOX to nuclear DNA [47]. Therefore, it is not surprising to state that DNA damage plays a role in early DOX-induced myocyte injury [71]. The level of antioxidant store of SOD, catalase and GPx enzymes within the cardiac tissues decreased in dose-dependent manner and correlated with the increasing DOX dose. A previous study proposed higher cardiac susceptibility to DOX cytotoxicity attributable to lower concentration of enzymatic defenses by super oxide dismutase-1 (SOD1) and catalase against free radical attack and a drug-related depression of cardiac glutathione peroxidase activity following exposure to doxorubicin [15]. It seems that mitochondria rely upon ATP to sustain contractile function, and consequently interference with this function is likely to cause the cardioselective toxicity [72]. However, the chronic phase in the present study showed a significant increase of cardiac biomarkers in comparison with the group-specific control, suggesting that mitochondrial redox status is not the dominant factor contributing to the observed mitochondrial damage. Interestingly, we noticed the presence of degranulated mast cells in the heart of chronic group, which aggravate the possible role of the bioactivated components of DOX [73].

## 5. Conclusions

Given the above information, 2 mg/kg body weight of DOX has shown a cardiotoxic effect in rats. The resulting induced acute toxic myocarditis is characterized by degenerative and necrotic changes in myocytes, mitochondrial damage, vacuolation, vascular changes, and occasionally inflammatory cell infiltration. These changes increase in severity with subsequent treatment with the same dose until reaching a cumulative dose of 6 mg/kg body weight over 15 days. On exposure to a cumulative dose of 8 mg/kg body weight with a withdrawal period of 15 days or a cumulative dose of 10 mg/kg body weight (cumulative therapeutic dose in man) with a withdrawal period of two or three months, predominant changes in chronicity were observed, such as disorganization and atrophy of myocytes, condensation and atrophy of mitochondria, and fibrosis with occasional focal necrosis, indicating incomplete elimination of DOX, and/or its metabolites. Future research seems warranted to explore more about the dose-dependent mechanisms of such induced toxicity of DOX that would help us in better understanding of the proper doses and resulting cardiomyopathy.

## Figures and Tables

**Table 1:** Serum cardiac enzymes and antioxidant biomarkers within the different animal groups.

Groups	AST (U/L)		CK–MB (U/L)		cTnT (µg/L)		Catalase (U/mg Protein)		SOD (mU/mg Protein)		GPx (mU/mg Protein)	
	Control (*n* = 3)	Treated (*n* = 5)	Control (*n* = 3)	Treated (*n* = 5)	Control (*n* = 3)	Treated (*n* = 5)	Control (*n* = 3)	Treated (*n* = 5)	Control (*n* = 3)	Treated (*n* = 5)	Control (*n* = 3)	Treated (*n* = 5)
G1	57.50 ± 3.12	74.80 ± 5.89 ^a^	304.40 ± 14.38	410.40 ± 9.40 ^a^	10.64 ± 2.12	16.85 ± 3.04 ^a^	57.32 ± 4.75	44.97 ± 4.56 ^a^	41.45 ± 2.34	31.96 ± 3.47 ^a^	29.11 ± 1.39	19.47 ± 2.64 ^a^
G2	55.30 ± 3.13	89.60 ± 6.39 ^a^	306.40 ± 22.33	532.40 ± 14.67 ^a^	10.70 ± 2.27	28.09 ± 2.18 ^a^	56.13 ± 4.75	36.08 ± 3.63 ^a^	40.37 ± 3.23	25.60 ± 3.62 ^a^	28.20 ± 1.30	19.21 ± 3.19 ^a^
G3	57.40 ± 2.43	130.00 ± 8.28 ^a,b,c^	309.38 ± 16.27	809.80 ± 32.87 ^a,b,c^	10.92 ± 1.75	40.34 ± 3.36 ^a,b,c^	53.22 ± 4.75	26.67 ± 2.83 ^a,b,c^	39.63 ± 2.20	22.02 ± 2.15 ^a,b,c^	28.62 ± 2.12	16.68 ± 2.58 ^a,b^
G4	58.50 ± 3.18	157.00 ± 5.66 ^a,b,c,d^	310.40 ± 23.11	900.20 ± 14.06 ^a,b,c,d^	11.36 ± 2.47	52.96 ± 3.66 ^a,b,c,d^	54.40 ± 4.75	20.05 ± 1.52 ^a,b^^,c^	40.34 ± 2.69	20.88 ± 4.38 ^a,b,c^	27.25 ± 1.04	12.93 ± 1.89 ^a,b,c^
G5	60.20 ± 2.27	117.20 ± 4.97 ^a,b,c,e^	311.38 ± 25.24	688.00 ± 19.95 ^a,b,c,e^	11.46 ± 2.23	38.54 ± 1.49 ^a,b,c,e^	56.62 ± 4.75	20.94 ± 3.45 ^a,b,c^	39.23 ± 2.34	15.12 ± 3.29 ^a,b,c,e^	28.75 ± 2.50	12.07 ± 2.88 ^a,b,c^
G6	63.80 ± 3.36	104.60 ± 7.44 ^a,b,c,d^	315.40 ± 21.74	610.80 ± 23.95 ^a,b,d^	12.13 ± 2.44	28.42 ± 2.42 ^a,b,d,e,f^	52.25 ± 4.75	32.76 ± 3.95 ^a,b,e,f^	38.14 ± 1.18	22.22 ± 2.96 ^a,b^	26.90 ± 1.18	14.54 ± 0.97 ^a,b^
G7	62.30 ± 3.38	89.80 ± 10.76 ^a,f,g^	321.38 ± 18.19	589.00 ± 11.58 ^a,f^	13.83 ± 2.26	22.38 ± 3.11 ^a,f^	50.13 ± 4.75	39.06 ± 2.37 ^a^^,f^	38.25 ± 3.16	30.50 ± 3.83 ^a,f^	25.85 ± 2.38	17.05 ± 2.05 ^a,f^

Data are expressed as mean ± SD. The significance level is indicated by superscript letters; “^a^” represents the statistical significance of comparison of the data in any treated group to the data in the group-specific control, “^b^” represents the statistical significance of comparison of the data in treated G2 to the data in treated G1, “^c^” represents the statistical significance of comparison of the data in treated G3 to the data in treated G2, “^d^” represents the statistical significance of comparison of the data in treated G4 to the data in treated G3, “^e^” represents the statistical significance of comparison of the data in treated G5 to the data in treated G4, “^f^” represents the statistical significance of comparison of the data in treated G6 to the data in treated G5 and “^g^” represents the statistical significance of comparison of the data in treated G7 to the data in treated G6.

## Data Availability

The data that support the findings of this study are available on request from the corresponding author.
